# Re: Saludares et al: A Phase I Randomized Trial of Topical Insulin for Glaucoma: Safety and Efficacy Outcomes (*Ophthalmol Sci.* 2026;6(2):101032)

**DOI:** 10.1016/j.xops.2026.101172

**Published:** 2026-03-30

**Authors:** Mohammadmehdi Hatami, Mohsen Zare

**Affiliations:** Department of Ophthalmology, Torfeh Medical Center, Shahid Beheshti University of Medical Sciences, Tehran, Iran

To the Editor:

Saludares et al's research may represent a new step toward developing intraocular pressure–independent treatments for glaucoma.^1^ Given the importance of the article, we have a few considerations and questions for the research team that may also be helpful for subsequent phases of the study.

1. Patients continued prior intraocular pressure–lowering topical treatments, and the worse eye was selected for insulin drops. Ultimately, the intervention group was reported to have higher retinal nerve fiber layer (RNFL) thickness interpreted as a potential neurotrophic or neuroprotective effect. However, a substantial proportion of cases had advanced glaucoma, as evidenced by mean deviation worse than –12 dB (Table 2 in the original article). The manuscript does not include raw RNFL thickness data for individual eyes. Given the severity of the patients' glaucoma, eyes with more severe baseline damage may have shown an attenuation in RNFL changes due to the “floor effect.”^2^ This could result in relative stabilization in RNFL thickness in treated eyes compared to fellow eyes, potentially confounding attribution to a true neuroprotective effect. We would like to know how the investigators ruled out the possible contribution of a floor effect to their observations. Would randomizing eyes, regardless of severity, help analyze the later phases of the study better?
2. The researchers reported that five versus zero eyes had a ≥5 μm increase in average RNFL thickness after 1 month, favoring the insulin group (*P*=0.0476). They also stated that the difference in RNFL thickness increase between the study groups was +1.11 μm versus +2.5 μm, favoring the insulin group. However, the test–retest variability of the Cirrus OCT device is reported to be approximately ±4 μm.^3^ While the categorical analysis mitigates this concern to some extent, the small average change remains within reported variability. Thus, these numbers should be interpreted with caution.
3. A history of surgery was not an exclusion criterion in this study. This phase was related to the safety of topical insulin. It is important to note that, despite numerous published reports on the effects of topical insulin on ocular surface diseases, our knowledge of its long-term effects on glaucoma patients is limited. Insulin promotes epithelialization and neuroregeneration at the corneal surface via insulin-like growth factor-1 receptor-mediated signaling.^4^ The current study similarly postulates an insulin-like growth factor-1 receptor–mediated neurotrophic effect on retinal ganglion cells. In patients who have undergone glaucoma surgery, such as trabeculectomy, similar mechanisms may lead to increased fibroblast growth or vascularization of the bleb, potentially increasing the risk of bleb fibrosis or failure. How have the investigators considered or addressed these potential complications?

Once again, we would like to thank the research team for their valuable effort and report. We look forward to reading their follow-up studies.

## References

[1] Saludares M., Wennberg-Smith Z., Beykin G. (2025). A phase 1 randomized trial of topical insulin for glaucoma: safety and efficacy outcomes. Ophthalmol Sci.

[2] Tomita R., Rawlyk B., Sharpe G.P. (2024). Progressive changes in the neuroretinal rim and retinal nerve fiber layer in glaucoma: impact of baseline values and floor effects. Ophthalmology.

[3] Wadhwani M., Bali S.J., Satyapal R. (2015). Test-retest variability of retinal nerve fiber layer thickness and macular ganglion cell-inner plexiform layer thickness measurements using spectral-domain optical coherence tomography. J Glaucoma.

[4] Stuard W.L., Titone R., Robertson D.M. (2020). The IGF/Insulin-IGFBP axis in corneal development, wound healing, and disease. Front Endocrinol (Lausanne).

